# Comparison of anesthetic effects of different doses of alfentanil combined with ciprofol in elderly patients undergoing ERCP: a randomized controlled trial

**DOI:** 10.1186/s12871-023-02325-4

**Published:** 2023-10-31

**Authors:** Jun Hu, Xiuxia Gu, Wenhao Zhu, Xiuli Zhu, Fanceng Ji, Yanhua Luo, Ning Liu

**Affiliations:** 1 Department of Anesthesiology, Weifang People’s Hospital, Weifang, 261000 China; 2 Department of Anesthesiology, Dongying Shengli Oilfield Central Hospital, Dongying, China

**Keywords:** Endoscopic retrograde cholangiopancreatography (ERCP), Alfentanil, Ciprofol

## Abstract

**Background and objects:**

Patients undergoing endoscopic retrograde cholangiopancreatography (ERCP) are often old and poor in physical fitness. The purpose of this study was to investigate the anesthetic effect of different doses of alfentanil combined with ciprofol in elderly patients undergoing endoscopic retrograde cholangiopancreatography (ERCP).

**Methods:**

In this clinical trial, 137 patients, who were candidates for ERCP were randomly divided into three groups. Group A were given 0.15 μg/kg/min of alfentanil in maintenance stage, Group B were given 0.25 μg/kg/min and Group C were given 0.35 μg/kg/min. Mean arterial pressure (MAP), heart rate (HR), oxygen saturation (SpO2) of the patients at each time point including the entry into the operation room (T0), at the beginning of surgery(T1), 10 min after surgery(T2), 20 min after surgery(T3), 30 min after surgery(T4),endoscopy withdrawal (T5) were recorded. Adverse events(including respiratory depression, body movement and hypoxemia),the dosage of ciprofol, the time of operation time and awakening were recorded.

**Results:**

Compared with Group A, MAP and HR in Group B and Group C was decreased during T1-T5 (*P* < 0.05). Compared with group B, MAP and HR in group C was decreased during T1-T5 (*P* < 0.05). Compared with Group A and Group C,the number of adverse reactions of Group B was decreased(*P* < 0.05). There was no statistical difference in surgical time among the three groups(*P* > 0.05),but a statistically significant difference in recovery time (*P* < 0.05).

**Conclusion:**

The adverse events of alfentanil 0.25μg/kg/min combined with ciprofol were low, and the anesthetic effect was the best.

## Introduction

ERCP is mainly used for the diagnosis and treatment of biliary and pancreatic diseases. The most commonly used anesthesia methods of ERCP are endotracheal intubation general anesthesia (GA) and deep sedation anesthesia(DSA) [1–3]. During the operation, the patient's side prone or prone position causes chest and abdomen compression, which has a significant impact on respiration and circulation [4, 5]. Research shows that [6, 7], although GA group can provide good airway protection during operation, the incidence of hypotension, acute respiratory failure and pneumonia is higher than that of non GA group. Another multicenter retrospective study report showed that the incidence of sedation related adverse events in patients with DSA (6%) was significantly higher than that in patients with GA (0.4%) [8]. In view of the challenges of ERCP anesthesia caused by the fragile hemodynamics and respiratory function of elderly patients, it is significant to explore a safe, simple and effective anesthetic method for elderly patients with DSA. This study aims to provide a new idea for clinical anesthetic drug optimization by observing the effectiveness and safety of different doses of alfentanil combined with ciprofol in ERCP.

## Methods

### Study design and ethics

The study was designed as an assessor-blinded parallel-group randomised controlled trial. This study was approved by the Ethics Committee of the Weifang People’s Hospital (KYLL20221128-1). And the study was registered in Chinese Clinical Trial Registry (02/12/2022; Chinese Clinical Trial registry, No.ChiCTR2200066366). Written informed consent was obtained from all participants. The trial report complies with the Consolidated Standards of Reporting Trials (CONSORT) checklist.

### Participants

Inclusion criteria: ① ERCP was performed at selected time; ② Age 65 ~ 85 years old, regardless of gender; ③ The American anesthesiologist association (ASA) is classified as II ~ III; ④ Depending on the disease, surgery includes: balloon dilatation lithotomy, basket lithotripsy and/or lithotomy, and endoscopic stent placement in the pancreas/bile duct. exclusion criteria:① morbid obesity, body mass index (BMI) > 30 kg/m^2^; ② Severe respiratory disease; ③ Long term history of taking psychotropic drugs and cognitive dysfunction; ④ The modified Malampati classification was Grade IV; ⑤ allergies to any drug involved in the study.All patients enrolled in this study were evaluated by anesthesiologists above the attending doctor before anesthesia and signed informed consent forms. If the patient refused to participate in the study or the surgical method was changed, the study was stopped. Te study protocol had no important harmful or unintended effects on participants.

### Outcomes

Observe and record the mean arterial pressure (MAP), heart rate (HR), and blood oxygen saturation (SpO_2_) before anesthesia induction (T0), at the beginning of surgery (T1), 10 min (T2), 20 min (T3), 30 min (T4) and at the end of surgery (T5). The adverse events of anesthesia during operation were recorded, including body movement reaction, hypoxemia (blood oxygen saturation below 93%), and hypotension (mean arterial pressure below 30%); Record the operation and recovery time.

### Randomisation and blinding

Patients were assigned to three groups A, B, and C using a random number table method.Participants and outcome assessors were blinded to group allocation, while anaesthesia providers could not be blinded because of the significant differences between the anaesthetic techniques.

### Procedures

The patient was deprived of food and water for 8 h before the operation. After entering the operating room, select the patient's right forearm vein for injection. The patient swallowed 10 ml of dyclonine hydrochloride mucilage. They were given an ECG monitor, connected to the bispectral index of the EEG, and then inhaled oxygen through a nasal catheter. Fully supply oxygen and remove nitrogen for 3 min under spontaneous ventilation, and the oxygen flow rate is 4–6 L/min. Anesthesia induction: three groups of patients were given alfentanil 5 μg/kg (slow injection within 30 s) and 0.4 ± 0.1 mg/kg (slow injection within 90 s) of ciprofol [3, 9]. If the bispectral index (BIS) of EEG drops to 60, the surgery will be started and the induced dose of ciprofol will be changed to the maintenance dose. Anesthesia maintenance: group A, B and C were treated with alfentanil μg/kg/min、0.25 μg/kg/min、0.35 μg/kg/min combined with 1.5 mg/kg/h ciprofol for continuous infusion (maintain BIS value between 50 and 60 according to BIS value adjustment). Blood pressure, ECG, SPO_2_ and BIS were monitored during operation. When the nasobiliary duct is placed at the end of the operation, drug infusion is stopped. All operations were performed by the same group of doctors and the same type of duodenoscopy. If the patient has physical activity during the operation, 0.1 mg/kg of ciprofol is given for treatment. If SpO_2_ < 93%, immediately raise the jaw, and if necessary, place the nasopharynx airway to help ventilate.

### Statistical analyses

**Fig.1:**
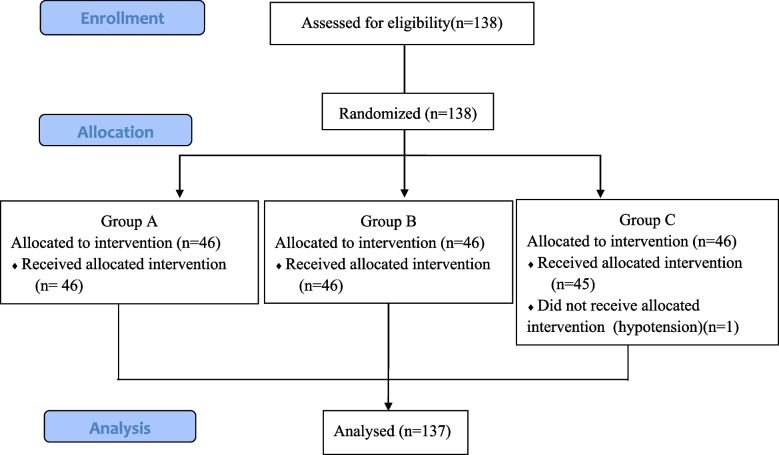
Patients enrollment diagram

Experimental data were processed by statistical product and service solutions (SPSS) 26.0. Data are summarized as the mean (standard deviation), median (interquartile range [IQR]), or number (%). Categorical data were analyzed with Fisher’s exact test or the X2 test. The measurement data adopts the analysis of repeated measure date ANOVA, One-way ANOVA and T-test. Two-sided *p* values < 0.05 were considered significant Fig. 1.

## Results

### Basic information of the research objects

Summarize and analyze the age, gender proportion, weight, ASA grades, operation time and the dosage of ciprofol in the three groups of patients. The comparison of the above information all showed no statistical differences(*P* > 0.05) Table 1.

**Table 1:** Comparison of basic information about the patients in three groups

Variables	GroupA(*n*=46)	GroupB(*n* = 46)	GroupC(*n* = 45)	*P* value
Age(year)	74.09 ± 6.40	74.28 ± 6.07	73.62 ± 6.63	0.879
Gender(M/F)	22/24	18/27	20/25	
Weight(kg)	65.98 ± 8.87	63.78 ± 81.46	65.62 ± 9.40	0.443
ASA score				0.439
II	31(67%)	29(63%)	31(69%)	
III	15(33%)	17(37%)	14(31%)	
Process time(min)	50.24 ± 12.50	48.07 ± 9.15	50.44 ± 10.50	0.508
Ciprofol dosage(mg)	107.62 ± 24.47	100.72 ± 18.85	97.77 ± 21.97	0.091

### Comparison of hemodynamics of the patients in the three groups at different time points

**Fig. 2:**
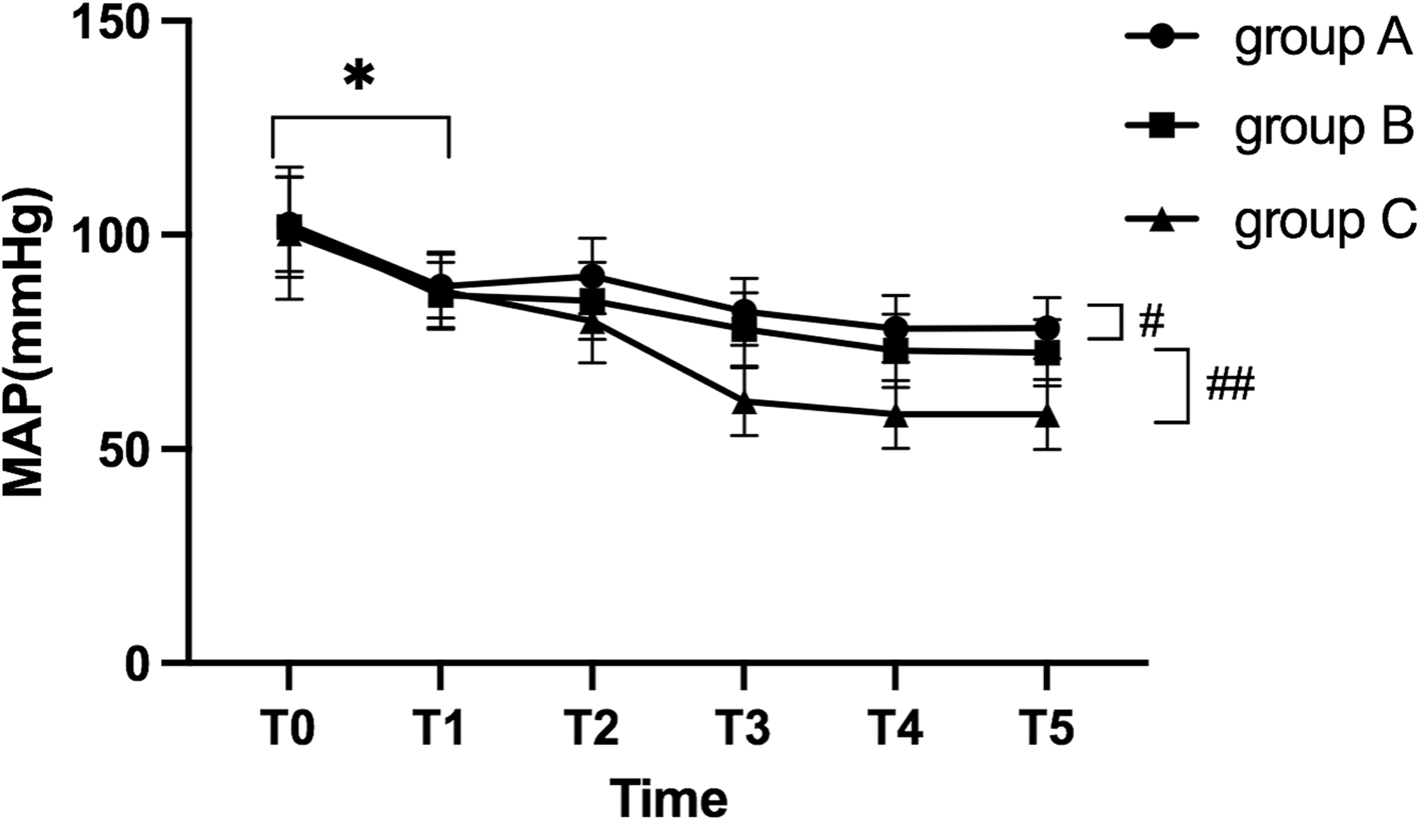
Comparison of MAP of the patients in three groups at different time points (*showed that the comparison with MAP at T0 and T1 had no statistical differences, (*P* > 0.05); # showed statistical difference of MAP between group A and group B from T1 to T5(*P* < 0.05); ## showed statistical difference of MAP between group B and group C from T1 to T5 (*P* < 0.05))

The comparison of MAP of the patients in the three groups at T0 and T1 showed no statistical differences (*P* > 0.05). From T1, MAP of the patients in the three groups showed a downward trend. Compared with group A, MAP in group B decreased from T1 toT5(*P* < 0.05). Compared with group B, MAP decreased in group C from T1 to T5 (*P* < 0.05) Fig. 2.

**Fig. 3:**
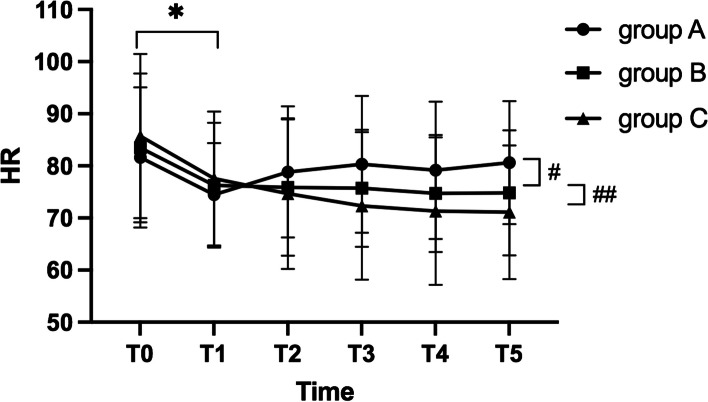
Comparison of HR of the patients in three groups at different time points (*showed that the comparison with HR at T0 and T1 had no statistical differences, (*P* > 0.05); # showed statistical difference of HR between group A and group B from T2 to T5(*P* < 0.05); ## showed statistical difference of HR between group B and group C from T2 to T5 (*P* < 0.05))

The comparison of HR of the patients in the three groups at T0 and T1 showed no statistical differences (*P* > 0.05). From T1, the HR in group A gradually increased with the stimulation of surgery. The HR in group B was relatively stable. HR in group C decreased significantly. Compared with group A, HR in group B decreased from T1 to T5 (*P* < 0.05). Compared with group B, HR decreased in group C from T1 to T5 (*P* < 0.05) Fig. 3.

### Comparison of adverse events in three groups of patients Table 2

**Table 2:** Comparison of adverse events in three groups of patients

group	body movement	hypoxemia	hypotension	total
group A	17(36.96%)*	1(2.17%)	4(15.38%)	22(15.94%)
group B	4(11.11%)#	1(2.17%)##	6(13.04%)##	11(8.00%)*
group C	2(4.44%)	12(26.67%)*	25(55.56%)*	39(28.89%)

^*^Compared with other groups, *p* < 0.05

^#^ Compared with group C, *p* > 0.05

^##^ Compared with group A, *p* > 0.05

The number of intraoperative body movements in group A, B and C were 17(36.96%),4(11.11%),2(4.44%), respectively. Compared with group A, the incidence of body movements in group B and group C were significantly lower (*P* < 0.05), and there was no statistical significance in the incidence of body movements in group B and group C (*P* > 0.05).

The incidences of hypoxemia in the three groups were 1(2.17%), 1(2.17%), 12(26.67%), respectively. Compared with group A and group B, the incidence of respiratory depression in group C was significantly higher ( *P* < 0.05).

The incidences of hypotension in the three groups were 4(15.38%), 6(13.04%), 25(55.56%), respectively. Compared with group A and group B, the incidence of hypotension in group C was significantly higher ( *P* < 0.05), The incidence of hypotension in group A and group B had no statistical differences ( *P* > 0.05).

### Comparison of operation time and awakening time in three groups

**Fig. 4:**
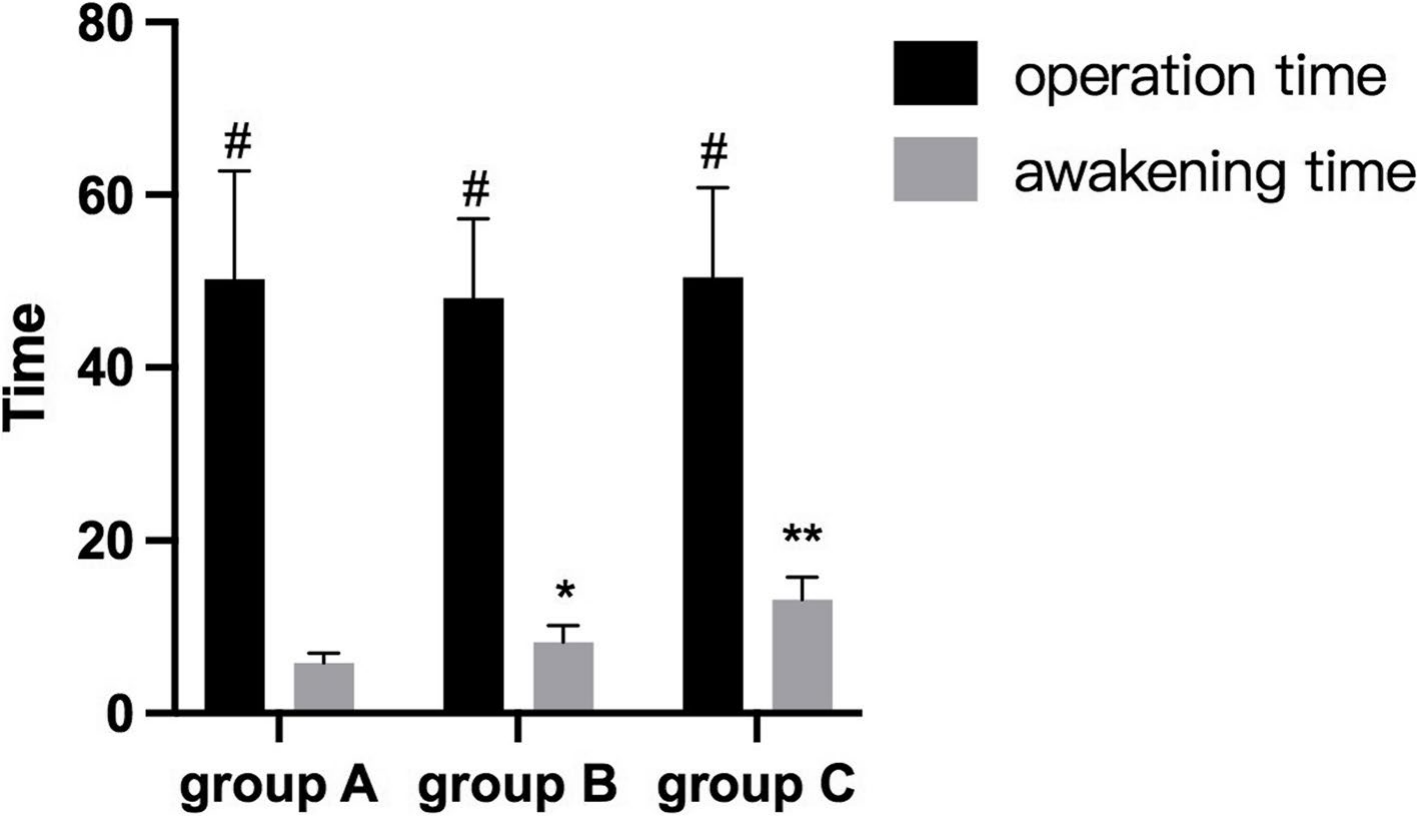
Comparison of operation time and awakening time (# showed that there were no statistical differences among the three groups, *P* > 0.05;*showed that the comparison with the group A and group B had statistical differences,(*P* < 0.05); **showed that the comparison with the group B and group C had statistical differences, (*P* < 0.05))

The comparison of operation time in the three groups showed no statistical differences(*P* > 0.05). The awakening time of the three groups of patients was 5.85 ± 1.12 min, 8.22 ± 1.96 min and 13.13 ± 2.59 min respectively. There was a significant difference between group A and group B (*P* < 0.05), and there was a significant difference between group B and group C (*P* < 0.05) Fig. 4.

## Discussion

During ERCP anesthesia, insufficient depth of anesthesia will lead to cough, body movement, hypertension and increased heart rate. If the depth of anesthesia is too deep, respiratory and circulatory inhibition will occur. In particular, intraoperative hypotension may cause organ damage in elderly patients, resulting in adverse postoperative results [10]. In this study, the dosage of alfentanil was divided into three dosage levels: small, medium and large. By combining with ciprofol, the patients were ensured to complete the operation safely, and the optimal dosage of alfentanil and the best continuous infusion dose of alfentanil in elderly ERCP Surgery was obtained.

Propofol is widely used for gastrointestinal endoscopy patients, but it has significant limitations, including individual diferences in pharmacokinetics and pharmacodynamics, respiratory depression, hypotension, and lack effective antagonists [11]. Ciprofol is a new intravenous anesthetic [12], belong to γ‐ Aminobutyric acid A receptor agonist has the same characteristics as propofol, such as rapid action, rapid onset, no accumulation, and rapid recovery. The incidence of injection pain and adverse respiratory events is significantly reduced compared with propofol [13]. Since the single use of ciprofol is not enough to relieve pain, the combination of ciprofol and intravenous opioids is used to play a synergistic role [14, 15], so as to improve the quality of anesthesia and enhance the antistress ability of patients [16, 17]. In this study, the BIS value reflecting the depth of anesthesia was used to guide the infusion of ciprofol [18], maintain BIS value between 50–60 [19]. To prevent the higher blood concentration caused by the higher dosage of ciprofol, which may lead to the excessive depth of anesthesia to interfere with the evaluation of the difference of different doses of alfentanil.

Alfentanil is widely used in intravenous general anesthesia. The combination of alfentanil and dexmedetomidine, ketamine or propofol has been well used in various short and minor operations [1, 20–22]. Previous studies on alfentanil mainly focused on the use of single bolus injection or on-demand administration of alfentanil [23]. In this study, continuous infusion can improve the quality of sedation and analgesia, rapidly adjust the depth of anesthesia, and significantly reduce the occurrence of hemodynamic fluctuations and respiratory depression. We found that the incidence of respiratory depression in the high dose group of alfentanil increased significantly, indicating that when the dosage of alfentanil reached 0.35μg/kg/min, the safety of respiration decreased significantly. In the anesthesia state, the heart rate will decrease due to sympathetic inhibition. We found that alfentanil has a strong effect on reducing heart rate, so the heart rate of patients in the high dose group of alfentanil decreased most significantly.

In the three experimental groups, group A had a higher incidence of body movements requiring the intervention of the surgeon, especially when the stimulus was strong (such as when the duodenal papilla or bile duct was stretched). The incidence of body movements requiring surgical intervention in group B and C was low. However, the incidence of deep breathing in group C patients was high, and the number of hypotension that needed to be treated increased, which had a certain impact on the operation of the surgeon. In comparison, surgeons were most satisfied with group B. In terms of recovery, group C took a long time. There were no complications such as delayed awakening, restlessness during awakening and disturbance of consciousness in the three experimental groups. Follow up after operation showed that no intraoperative awareness occurred in all patients.

### Limitations

During ERCP, duodenal peristalsis or spasm is a headache matter. Through observation, it is found that there is no difference in duodenal peristalsis or spasm among the three groups of patients during surgery. In order to solve this problem, further research is needed.

## Conclusion

Alfentanil 0.25 μg/kg/min dose group with ciprofol can be safely and effectively used for ERCP anesthesia in elderly patients.The incidence of overall adverse events (respiratory depression, body motion reaction, times of hypotension) in this group was lowest, and the anesthetic effect was the best.

## Data Availability

The datasets used and analyzed during the current study are available from the corresponding author on reasonable request. Ning Liu e-mail: guyueyinghui@163.com.
